# Artificial Intelligence and Machine Learning in Spine Research: A New Frontier

**DOI:** 10.3390/bioengineering11090915

**Published:** 2024-09-13

**Authors:** Min Cheol Chang

**Affiliations:** Department of Physical Medicine and Rehabilitation, College of Medicine, Yeungnam University, 42415, Daemyungdong, Namku, Taegu 705-717, Republic of Korea; wheel633@gmail.com

## 1. Introduction

Artificial Intelligence (AI) refers to the creation of computer systems capable of performing tasks typically requiring human intelligence [1], such as problem-solving, decision-making, language comprehension, perception, and learning. Different from systems that merely execute predefined commands, AI systems can learn directly from vast datasets and make autonomous decisions [1]. AI aims to simulate human cognitive functions, enabling machines to learn from data, adapt to new information, and make predictions or decisions without explicit programming for specific tasks. Machine Learning (ML)—a subset of AI—focuses on developing algorithms that allow computers to learn from data and improve their performance over time without being explicitly programmed [2]. ML models identify patterns in data and make predictions or decisions based on those patterns, enabling machines to learn and generalize from examples and increase their accuracy with more exposure to information.

AI and ML are transforming numerous industries, and healthcare is no exception [3]. In spine research, AI and ML are proving to be powerful tools for improving diagnostics, optimizing treatment plans, and enhancing patient outcomes. These technologies can analyze large quantities of data quickly and accurately, revealing patterns that are often imperceptible to humans [4]. This editorial presents some examples of how AI or ML is being applied in spine research.

## 2. The Role of AI and ML in Spine Research

Traditionally, spine research depends on manual and time-consuming methods for data collection, analysis, and interpretation. Moreover, using traditional statistical methods limits the ability to analyze and process imaging data effectively [1,2]. However, the emergence of AI and ML has revolutionized these processes [1,2]. Researchers can now leverage algorithms to analyze imaging data, recommend personalized treatment strategies, and even predict patient outcomes [5,6,7,8]. This advancement is especially significant in spine research, where the complexity of spinal disorders and variability in patient responses often complicate treatment planning.

## 3. AI and ML in Analyzing Imaging Data

AI and ML are making significant advances in the analysis of medical imaging within spine research. Imaging technologies, such as magnetic resonance imaging (MRI), computed tomography (CT), and X-rays, are essential for evaluating spinal conditions and guiding treatment plans for patients with spinal disorders [9]. However, interpreting these images can be subjective, time-consuming, and susceptible to human error.

AI and ML algorithms enhance image analysis by identifying subtle patterns and abnormalities that may be overlooked by the human eye. These technologies not only improve diagnostic accuracy but also accelerate the process. For instance, AI models can automatically segment spinal structures from imaging data, aiding clinicians in detecting spinal pathologies such as spinal stenosis, herniated discs, or spinal tumors with greater precision [7,10,11]. Hallinan et al. developed an AI model for the automated detection and classification of lumbar central canal, lateral recess, and foraminal stenosis [11]. They showed significant agreement between their AI model and radiologists in classifying stenosis severity, achieving kappa values of 0.98, 0.98, and 0.96 for the central canal stenosis, 0.92, 0.95, and 0.92 for lateral recess stenosis, and 0.94, 0.95, and 0.89 for foraminal stenosis. Gilberg et al. developed an AI algorithm to detect metastatic lesions in abdominal and thoracic CT scans [10]. In their study, the AI algorithm exhibited a sensitivity of 75.0% in identifying potentially malignant spinal bone lesions. Moreover, it improves the sensitivity of the radiologist in detecting metastasis by 20.8 percentage points.

Additionally, AI and ML algorithms could predict disease progression [12]. For example, by analyzing historical imaging and clinical data, algorithms can predict the likelihood of spinal degeneration or scoliosis progression. This predictive capability enables clinicians to make more informed treatment decisions for each patient and potentially prevent conditions from worsening before they become critical.

## 4. AI and ML in Personalized Treatment Planning

Personalized medicine is one of the most promising applications of AI and ML in spine research. The spine of each patient and their response to different treatments is unique. AI-driven models can analyze extensive datasets, including demographics, clinical history, genetic information, and imaging data, to predict how a patient might respond to specific treatments.

ML algorithms can determine which surgical techniques are most likely to succeed based on the unique profile of a patient [13]. Factors influencing spine surgery outcomes include the type of spinal disorder, patient anatomy, and surgeon experience [14,15]. AI-based systems can provide surgeons with a comprehensive analysis of these factors, helping them to select the most appropriate surgical approach. This can lead to fewer complications, faster recovery times, and improved patient satisfaction.

Furthermore, robotic-assisted spine surgery is another area where AI and ML are advancing rapidly [16]. These systems can improve the precision of surgical procedures by providing real-time feedback based on preoperative imaging and predictive models [16]. Although fully autonomous surgeries remain experimental, the ongoing evolution of AI may allow this capability in the future.

## 5. AI and ML in Predicting Therapeutic Outcomes

Another significant advantage of AI and ML in spine research is their ability to predict patient outcomes. ML models can forecast recovery times, potential complications, and therapeutic outcomes for different treatment approaches by analyzing clinical and imaging data from patients with spinal disorders [17,18,19,20,21]. These insights enable clinicians and patients to make more informed decisions regarding treatment options.

For instance, in spinal surgery, ML algorithms can predict the likelihood of reoperation or complications such as infections or hardware failure. These predictive tools are essential for preoperative planning, helping surgeons mitigate risks and tailor interventions to individual patients [17,18,20]. Beyond surgical outcomes, AI and ML can also predict the effectiveness of nonsurgical outcomes such as spine intervention [19,21]. Kim et al. developed an AI algorithm that predicts therapeutic outcomes following transforaminal epidural stenosis injection (TFESI) for managing chronic lumbosacral radicular pain caused by herniated lumbar discs, using T2-weighted sagittal lumbar spine MRI data [19]. A “good outcome” was defined as a ≥50% reduction in pretreatment pain after 2 months, while a “poor outcome” was defined as a <50% pain reduction after the same duration. In the prediction of therapeutic outcomes (good outcome vs. poor outcome) after TFESI on the validation dataset, the area under the curve was 0.827. Similarly, Wang et al. created an AI algorithm to predict the therapeutic outcome of cervical TFESI in patients with cervical foraminal stenosis using cervical axial MRI data [21]. The area under the curve of our developed model for predicting the therapeutic outcome of cervical TFESI in patients with cervical foraminal stenosis was 0.801.

AI-powered predictive analytics can help clinicians choose the most appropriate treatment paths, ultimately improving patient satisfaction and quality of life.

## 6. Challenges and Ethical Considerations

AI and ML hold great promise in spine research; however, they also present significant challenges. A major concern is the quality and quantity of data used to train these algorithms. In many cases, spine research involves small datasets, which can reduce the accuracy and generalizability of AI models. Furthermore, data bias presents a serious risk as certain populations may be underrepresented in clinical trials and studies. Training AI models on biased data may result in biased outcomes.

Another challenge is the integration of AI into clinical practice. Although AI can assist in decision-making, clinicians should maintain oversight and responsibility for patient care. AI should complement, not replace, human expertise. To ensure safe implementation, AI systems should be transparent and explainable, allowing clinicians to understand the rationale behind AI-generated recommendations.

Ethical issues should be considered, particularly regarding patient privacy and data security [22]. AI and ML rely on large amounts of patient data, raising concerns about how these data are collected, stored, and used. Regulatory frameworks need to evolve to address these issues and safeguard patient rights in AI-driven healthcare.

## 7. The Future of AI and ML in Spine Research

The future of AI and ML in spine research is promising. As these technologies evolve, we can anticipate increasingly sophisticated applications in diagnostics, treatment planning, and outcome prediction. Advances in deep learning, natural language processing, and computer vision will possibly lead to further breakthroughs in this field.

Moreover, the integration of AI and ML with other emerging technologies, such as wearable devices and telemedicine, may transform how spine conditions are monitored and managed. For instance, wearable sensors could continuously track the posture and movement of a patient, feeding this information into AI algorithms to deliver real-time feedback and personalized interventions. Telemedicine allows physicians to access AI-generated analysis and feedback, enabling patients to receive care without meeting physicians in person.

In the future, AI and ML may pave the way for more proactive and preventive spine care. These technologies could facilitate earlier interventions by identifying early signs of spinal degeneration or injury, reducing the need for invasive procedures, and improving overall patient outcomes.

## 8. Conclusions

AI and ML are transforming spine research in unprecedented ways. These technologies are enhancing diagnostics, personalizing treatments, and predicting outcomes, creating new opportunities for clinicians and researchers. However, their integration into clinical practice requires careful consideration of ethical issues, data quality, and clinician oversight. Undoubtedly, these technologies will play a pivotal role in shaping the future of spine research and care. In this Special Issue, “Artificial Intelligence and Machine Learning in Spine Research,” we explore the current applications of AI and ML in this field. This issue may advance spinal research and assist researchers in identifying promising new avenues for investigation.
